# Kids save lives: Who should train schoolchildren in resuscitation? A systematic review

**DOI:** 10.1016/j.resplu.2024.100755

**Published:** 2024-08-29

**Authors:** A. Mollo, S. Beck, A. Degel, R. Greif, J. Breckwoldt

**Affiliations:** a Institute of Anesthesiology, University Hospital Zurich, University of Zurich, Zurich, Switzerland; b Department of Internal Medicine, Spital Limmattal, Zurich, Switzerland; c Department of Intensive Care Medicine, Hamburg-Eppendorf University Medical Centre, Hamburg, Germany; d Department of Cardiology, Angiology and Intensive Care Medicine, Deutsches Herzzentrum der Charité, Hindenburgdamm 30, 12203 Berlin, Germany; e University of Bern, Bern, Switzerland; f Department of Surgical Science, University of Torino, Torino, Italy

**Keywords:** Systematic review, Resuscitation training, Basic life support, Schoolteachers, Peer-teaching, Kids-save-lives education

## Abstract

**Aim:**

CPR training for schoolchildren to increase bystander CPR-rates is widely applied. HCPs are regarded as the instructor gold standard, but using non-HCP instructors (e.g., peer-tutors, schoolteachers, medical students) challenges that. This systematic review assesses whether cardiopulmonary resuscitation (CPR) training for children led by peer-tutors, schoolteachers, or medical students results in different learning outcomes to training by health-care professionals (HCPs).

**Methods:**

We searched studies that compared CPR training for schoolchildren *(population)* delivered by peer-tutors, schoolteachers, or medical students *(intervention),* with training led by HCPs *(comparison),* assessing student knowledge, skills, willingness and/or confidence to perform CPR *(outcome)*. We included randomized and non-randomized controlled trials *(study design).* Medline, Embase, Psychinfo, Cinahl, Cochrane, Scopus, Web of Science, and Eric were searched from inception until December 23rd, 2023 *(timeframe)*. Two independent reviewers performed title, abstract, full text screening, bias assessment, and grading of certainty of evidence. We followed the Preferred Reporting Items for a Systematic Review and Meta-Analysis (PRISMA) guidelines, and registered the review with PROSPERO.

**Results:**

Of 9′092 studies identified, 14 were included. Comparison of intervention groups to HCP-led training showed similar overall results (knowledge, skills, self-confidence). Superior results for HCP training were only reported for ‘ventilation volume’, while schoolteachers and medical students achieved superior knowledge transfer. A meta-analysis was possible for ‘compression depth’ between peer-tutors and HCPs showing no significant differences. Certainty of evidence was ‘low’ to ‘very low’.

**Conclusion:**

This systematic review of CPR training for school children revealed that peer-tutors, schoolteachers and medical students achieve similar educational outcomes compared to those of HCPs. Non-HCPs training schoolchildren is an appropriate cost-efficient alternative and easy to implement in school curricula.

## Introduction

Outcome from out-of-hospital cardiac arrest (OHCA) strongly depends on layperson bystander cardiopulmonary resuscitation (CPR).^1^ CPR before the arrival of emergency services (EMS) can more than double survival rates.^2^, ^3^, ^4^, ^5^, ^6^, ^7^ Layperson CPR-training is associated with increased cardiac arrest recognition^8^ and higher likelihood of initiating CPR before EMS-arrival.^7^, ^9^ One way to achieve wide-spread CPR-training independent of socio-economic factors is to train schoolchildren.^10^ The ‘Kids-Save-Lives’ programme has been endorsed by the WHO since 2012^11^ and was highlighted in a statement by the ‘International Liaison Committee on Resuscitation’ (ILCOR) in 2023.^12^, ^13^ Overall, regular CPR training for schoolchildren has been shown to generate and consolidate knowledge and skills in all age groups.^12^

While only a few countries have implemented mandatory training of CPR for schoolchildren, most of these programmes have been established on a voluntarily basis. These programmes suffer mainly from two often-quoted barriers: (a) difficulties integrating the programmes into school curricula and scheduling problems, and (b) limitations of resources, namely expenses for manikins and for instructors.^14^, ^15^ Certified health care professionals (HCP) are considered the ‘gold standard’ for CPR instructors, but they pose scheduling challenges within a school curriculum and produce extra costs. As an alternative, CPR training in schools delivered by non-HCP instructors (medical students, schoolteachers, peer-tutors, or combinations thereof) offer easier integration into school curricula at lower cost. 16, 17, 18. The ILCOR Scientific Statement suggests using schoolteachers for training, however, this suggestion has not been based upon a rigorous systematic review.^12^ Therefore, the aim of this study was to systematically assess the existing literature comparing the effect of HCP-led CPR training to CPR training by medical students, schoolteachers, or peer-tutors (‘alternative instructors’). We did not address other alternatives to HCP-led training (such as self-learning) to keep the focus on the question ‘who should instruct’.

## Methods

This systematic review was registered at the Prospective Registry for Systematic Reviews (PROSPERO CRD42024491922) and followed the Preferred Reporting Items for a Systematic Review and Meta-Analysis (PRISMA).^19^

We organized the research question according to the PICOST format *(population, intervention, comparator, outcome, study design, timeframe).*

• Do schoolchildren *(population),*
• receiving CPR-training by peer-tutor, schoolteacher, or medical students *(intervention)*
• compared to those receiving CPR-training led by HCPs *(comparator)*
• show equivalent educational outcomes (knowledge, skills, willingness and/or confidence to perform CPR) after the CPR-training *(outcome)*?
• Peer-reviewed randomized controlled trials (RCTs) and non-randomized controlled studies (non-RCTs) were eligible *(study design)*.
• Publications from inception of each database to the search date (23.12.2023) were included. No language limitations were set as long as an English abstract was available *(timeframe)*.

### Definitions

As ‘peer-tutors’ we defined schoolchildren either of the same age as their ‘trainees’ or older. In all cases, the more precise wording would be ‘near’-peers as the peers have some kind of preceding training and additional knowledge before their teaching task. ‘HCP instructor’, was defined as any person with a professional background in health care with some kind of an instructor qualification.

### Selection of eligible articles

The search strategy was generated and conducted by an information specialist of the University of Zurich (Appendix A). We searched Medline, Embase, Psychinfo, Cinahl, Cochrane, Scopus, Web of Science and Eric. Finally, the reference lists of the included studies were screened by the first author for eligible studies that might have been missed by the search strategy. The inclusion criteria were: I) CPR training of schoolchildren, II) intervention group was trained by either peer-tutors, schoolteachers or medical students and III) the control group was instructed by HCPs. Exclusion criteria were: I) children’s median age >16 years, II) No control group, or control group that did not receive any training and III) Conference abstracts. For abstract screening, full text screening and the data collection processes, we used the software ‘Covidence’ [https://www.covidence.org]. All sources were screened by the first author and one additional author independently and conflicts were solved bilaterally through open discussion. In case of remaining discrepancies, a third reviewer was involved to achieve consensus. Data were extracted into Excel [https://www.microsoft.com/] sheets and comprised training formats, characteristics of schoolchildren, instructor training, and outcomes after training, including knowledge, skills, willingness to perform CPR and self-confidence at different post-intervention time points. For papers reporting graphic output only, we approximated the results based on the graphics.

Two independent reviewers conducted the risk of bias assessment with the Cochrane’s ‘Risk Of Bias 2′ (ROB2)-assessment for RCTs and the ‘Risk Of Bias In Non-randomised Studies – of Interventions’ (ROBIN-I) tool for non-RCTs at outcome level.^20^, ^21^ Authors of this review who were authors of eligible studies were excluded from assessment. Conflicts were resolved bilaterally through open discussion. Certainty of evidence was evaluated using the ‘Grading of Recommendations Assessment, Development and Evaluation’ (GRADE) tool.^22^

### Synthesis methods & effect measures

We grouped the studies according to the instructors’ professional backgrounds and according to post-intervention outcome time points. Outcome assessment included short-term (end-of-course), mid-term (later than end-of-course, up to 12 months), and long-term learning (>12 months). Following the ‘Synthesis Without Meta-Analysis’ (SWiM) reporting guidelines, we created *effect direction plots* showing whether the study reported significant differences between the intervention and control group.^23^

For pooled studies reporting the skills “chest compression rate and depth” in suitable metrics, we tested homogeneity with the Cochrane’s Q test.^24^ When there was sufficient homogeneity, we calculated the effect size using inverse-variance random-effects models comparing standardized mean differences for continuous data and odds ratio for binary data (for studies reporting % of children passing the skills test). Due to methodological differences between study groups, it was not feasible to perform meta-analyses for the outcomes ‘knowledge’ or ‘willingness to perform CPR’ and ‘confidence’. Meta-analysis was performed with SPSS (IBM, version 29.0 for Mac). Results are presented as forest plots. A confidence interval of α = 0.05 was used for both homogeneity testing and effect size calculation.

## Results

**Fig. 1:**
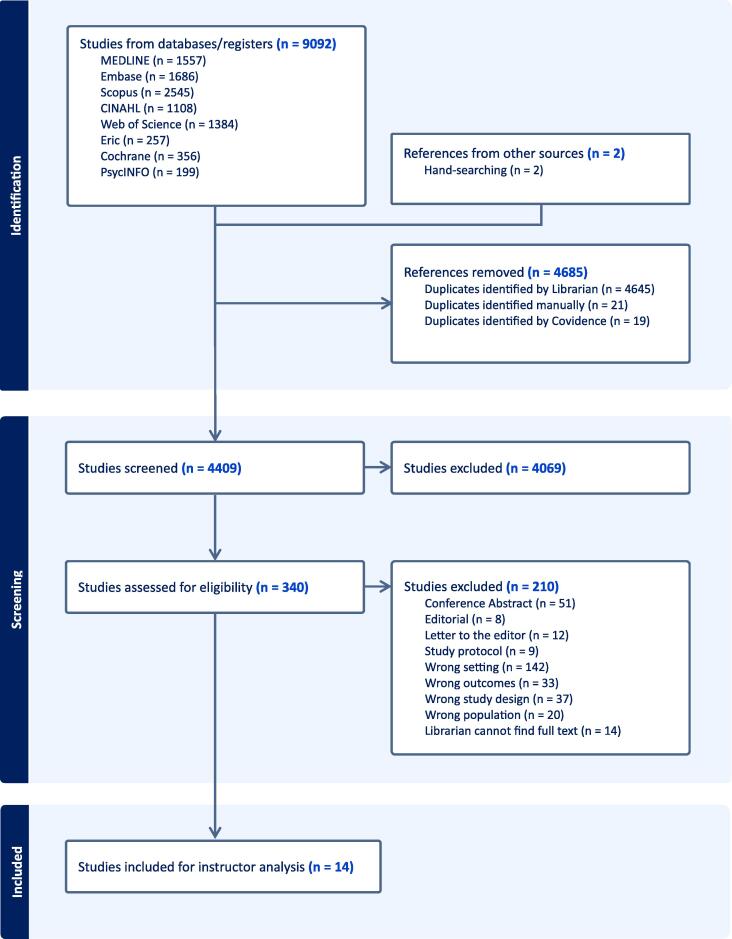
Prisma Flow Diagram.

The search identified 9′092 articles, and two additional studies were found by hand search. After removing 4′685 duplicates, 4′409 articles were included into the title and abstract screening, and 340 full texts were assessed, of which 14 eligible studies were included in the final analysis (PRISMA Flow chart Fig. 1, Study characteristics Table 1).

**Table 1:** Characteristics of included studies. RCT = randomized controlled trial, cRCT = cluster-randomized controlled trial, non-RCT = non-randomized experimental study. I = Intervention group, C = control group, yr = years.

**Author, Year, Country**	**Study Design**	**Analyzed sample size**	**Intervention**	**Time of outcome**	**Outcomes**	
Beck, 2015, Germany ^25^	RCT	I: 471, C: 466	Peer-led training	End-of-course	Skills	*Skill assessment using a checklist*
Bohn, 2012, Germany ^30^	non-RCT	I: 78, C: 66	Schoolteacher-led training	6–12 months; ≥ 12 months (2 yr, 3 yr, 4 yr)	Skills, knowledge, self-confidence	*CPR data registered by manikin.* *Knowledge and self-confidence by questionnaire.*
Choi, 2015, Korea ^26^	non-RCT	I: 119, C: 68	Peer-led training	≤3 months	Knowledge, Willingness	*Questionnaire*
Cuijpers, 2016, Netherlands ^31^	RCT	I1: 50, I2: 53, C: 41	I1: schoolteacher-led training; I2: medical students led training	End-of-course; ≤3 months	Skills	*Skill assessment using a checklist (Cardiff Test).* *CPR data registered by manikin.*
Damvall, 2022, Norway ^27^	non-RCT	I: 982, C: 76	Peer-led training	End-of-course; 6–12 months	Skills	*CPR Data registered by manikin.*
Dîrzu, 2018, Romania ^37^	cRCT	I: 64, C: 64	Medical students led training	≤3 months	Skills, Knowledge	*CPR data measured by manikin.* *Knowledge by questionnaire.*
Haseneder, 2019, Germany ^38^	cRCT	I: 193, C: 192	Medical students led training	≤3 months; 6–12 months	Knowledge and Self-confidence	*Questionnaire*
Jimenez-Fabrega, 2009, Spain ^36^	non-RCT	I: 219, C: 223	Schoolteacher-led training	End-of-course; 6–12 months	Knowledge	*Questionnaire*
Lanzas, 2022, Portugal ^32^	non-RCT	I: 199, C: 121	Schoolteacher-led training	End-of-course; ≤3 months	Skills, Knowledge	*Skill assessment using a checklist.* *Compression rates counted manually.* *Knowledge by questionnaire.*
Lukas, 2016, Germany ^35^	non-RCT	I: 99, C: 78	Schoolteacher-led training	6–12 months; >12 months (3 yr, 6 yr)	Skills, Knowledge, Self-confidence	*CPR data measured by manikin.* *Knowledge and confidence by questionnaire.*
Perez-Bailon, 2023, Spain ^33^	non-RCT	I: 327, C: 322	Schoolteacher-led training	End-of-course	Skills	*Skill assessment using a checklist*
Sabihah, 2020, Malaysia ^28^	RCT	I: 18, C: 18	Peer-led training	End-of-course; ≤3 months	Skills, Knowledge	*Skill assessment using a checklist.* *Knowledge by questionnaire.*
Santomauro, 2018, Italy ^29^	non-RCT	I: 164, C: 156	Peer-led training	End-of-course	Skills	*Skill assessment using a checklist.* *CPR data registered by manikin.*
Yeung, 2023, Hong Kong ^34^	RCT	I: 161, C: 150	Schoolteacher-led training	End-of-course; 6–12 months	Skills, Knowledge, Willingness	*Skill assessment using a checklist (only follow-up). Knowledge and willingness by questionnaire.*

Five studies analysed training by peer-tutors,^25^, ^26^, ^27^, ^28^, ^29^ seven by schoolteachers,^30^, ^31^, ^32^, ^33^, ^34^, ^35^, ^36^ and three by medical students.^31^, ^37^, ^38^ One study separately assessed both schoolteacher-led and medical student-led training, and therefore the study was included in both subgroups.^31^ The included studies were performed in Europe^25^, ^27^, ^29^, ^30^, ^31^, ^32^, ^33^, ^35^, ^36^, ^37^, ^38^ or Asia.^26^, ^28^, ^34^ Seven studies were RCTs, and seven were non-RCTs (prospective cohort studies). One follow-up study^35^ of a previous study^30^ (cohort overlap: 81.4%) was included as it provided additional data up to six years post-intervention. From another study comparing medical students with residents and emergency physicians,^37^ we only included the emergency physicians as HCPs. This study regarded the ‘resident’ group as a different competency level for training, and we wanted clear differences between intervention and control.

Children in all studies included were attending secondary school (age range: 10–18 years, average of the 7 RCTs^25^, ^29^, ^31^, ^33^, ^36^, ^37^, ^38^ was 14.4 years, median of the 7 non-RCTs^30^, ^32^, ^34^, ^27^, ^28^ was 15 years). At end-of-courses, 1635 children had been trained by their peers (control group *n* = 716), 956 by their schoolteachers (control group *n* = 857), and 53 by medical students (control group *n* = 41). Table 2 shows children included in the studies over time, whereas Supplemental Table 1 gives an overview of the durations of the training, the educational strategies used, and the instructors’ training.

**Table 2:** Students included in studies pooled by intervention groups. Time of outcome: T1: end-of-course, T2: ≤3 months, T3: 6–12 months, T4: 2 years T5: 3 years, T6: 4 years, T7: 6 years. *: overlapping cohort of I = 99 and C = 78. I = Intervention group, C = C.

**Intervention group**	**Number of studies for T1-T7**	**Number of recruited students**	**Number of trained students**	**Number of students analyzed at T1-T7**
**Peer-led training**	*T1: n = 4*^25^, ^27^, ^28^, ^29^ *T2: n = 2*^26^, ^28^ *T3: n = 1*^27^	4177	4145	T1: I = 1635, C = 716 T2: I = 137, C = 86 T3: I = 259, C = 11
**Schoolteacher-led training**	*T1: n = 5*^32^, ^33^, ^34^ *T2: n = 2*^31^, ^32^ *T3: n = 4*^30^, ^34^, ^35^, ^36^ *T5: n = 2*^30^, ^35^ *T4&T6: n = 1*^30^ *T7: n = 1*^35^	3475	2432	T1: I = 956, C = 857 T2: I = 209, C = 162 T3: I = 429, C = 422 * T4 & T6: I = 78, C = 66 T5: I = 177, C = 144 * T7: I = 99, C = 78
**Medical students led training**	*T1: n = 1*^31^ *T2: n = 3*^31^, ^37^, ^38^ *T3: n = 1*^38^	850	793	T1: I = 53, C = 41 T2: I = 310, C = 297 T3: I = 178, C = 179

Knowledge and self-confidence were assessed by questionnaires. Skill-assessments was performed either by using checklists 25, 28, 29, 31, 32, 33, 34 or by measuring CPR data by the manikin software (for details, see Table 1).^27^, ^35^, ^30^, ^31^

### Risk of bias and certainty of evidence

Risk of bias was rated from ‘low’ to ‘some concerns’ for RCTs, and ‘moderate’ to ‘serious’ for non-RCTs (Table 3.). The overall certainty of evidence was downgraded to ‘low’ or ‘very low’ due to risk of bias, inconsistency, and imprecision (supplemental Table 2).

**Table 3:** Risk of bias assessment. A: ROB-2-Tool («Risk Of Bias for randomized trials”) for RCTs, B: ROBINS-I-Tool («Risk Of Bias in Non-randomized Studies – of Interventions») for non-RCTs.

**Table 4:** Effect direction plots depicting comparisons between the intervention and control group for A: peer-tutors, B: schoolteacher-led training and C: medical students led training. ⇔: No significant difference,⇑/⇓:significant difference. EMS = emergency services, C = compression, AED = automatic external defibrillator, V = ventilation, Vol. = volume, mths = months, yrs = years.

**A)**																			
**Peer teaching**																			
		**Skills**															**Knowledge**	**Willingness**	**Confidence**
		Safety	Check response	Check breathing	Call EMS	C: Overall	C: Start	C: Depth	C: Rate	C: Position	C: Recoil	C: No flow time	AED	V: Rate	V: Vol.	Overall skills			
**End-of-course**	*Beck*^25^																		
	*Damvall*^27^																		
	*Sabihah*^28^																		
	*Santo-mauro*^29^																		
**≤3 months**	*Choi*^26^																		
	*Sabihah*^28^																		
**6**–**12 months**	*Damvall*^27^																		

**B)**																			
**teacher**																			
		**Skills**															**Knowledge**	**Willingness**	**Confidence**
		Safety Check	Check response	Check breathing	Call EMS	C: Overall	C: Start	C: Depth	C: Rate	C: Position	C: Recoil	C: No flow time	AED	V: Rate	V: Vol.	Overall			
**End-of-course**	*Cuijpers*^31^	*approach sequence:*																	
	*Jimenez-Fabrega*^32^																		
	*Lanzas*^33^																		
	*Pérez-Bailòn*^34^																		
	*Yeung*^35^																		
**<3 mths**	*Cuijpers*^31^	*approach sequence:*																	
	*Lanzas*^33^																		
**6**–**12 months**	*Bohn*^30^																		
	*Jimenez-Fabrega*^32^																		
	*Lukas*^38^																		
	*Yeung*^35^																		
**2 yrs**	*Bohn*^30^																		
**3 yrs**	*Bohn*^30^																		
	*Lukas*^38^																		
**4 yrs**	*Bohn*^30^																		
**6 yrs**	*Lukas*^38^																		

**C)**																			
**med student**																			
		**Skills**															**Knowledge**	**Willingness**	**Confidence**
		Safety Check	Check response	Check breathing	Call EMS	C: Overall	C: Start	C: Depth	C: Rate	C: Position	C: Recoil	C: No flow time	AED	V: Rate	V: Vol.	Overall			
**End-of-course**	*Cuijpers*^31^	*approach sequence:*																	
**<3 mths**	*Cuijpers*^31^	*approach sequence:*																	
	*Dîrzu*^36^																		
	*Haseneder*^37^																		
**6**–**12 months**	*Haseneder*^37^																		

### Peer-tutors compared to training led by HCP (*n* = 5) (Table 4A)

Four studies assessed overall skills at short-term^28^, ^29^, mid-term^28^, and long-term, ^27^ showing no significant differences between peer-led and expert led groups. However, one study reported significantly inferior outcomes for peer-led training (short- and mid-term assessments) for the parameters ‘check response and breathing’, ‘call EMS’, ‘compression depth’ and ‘compression recoil’.^28^

**Fig. 2:**
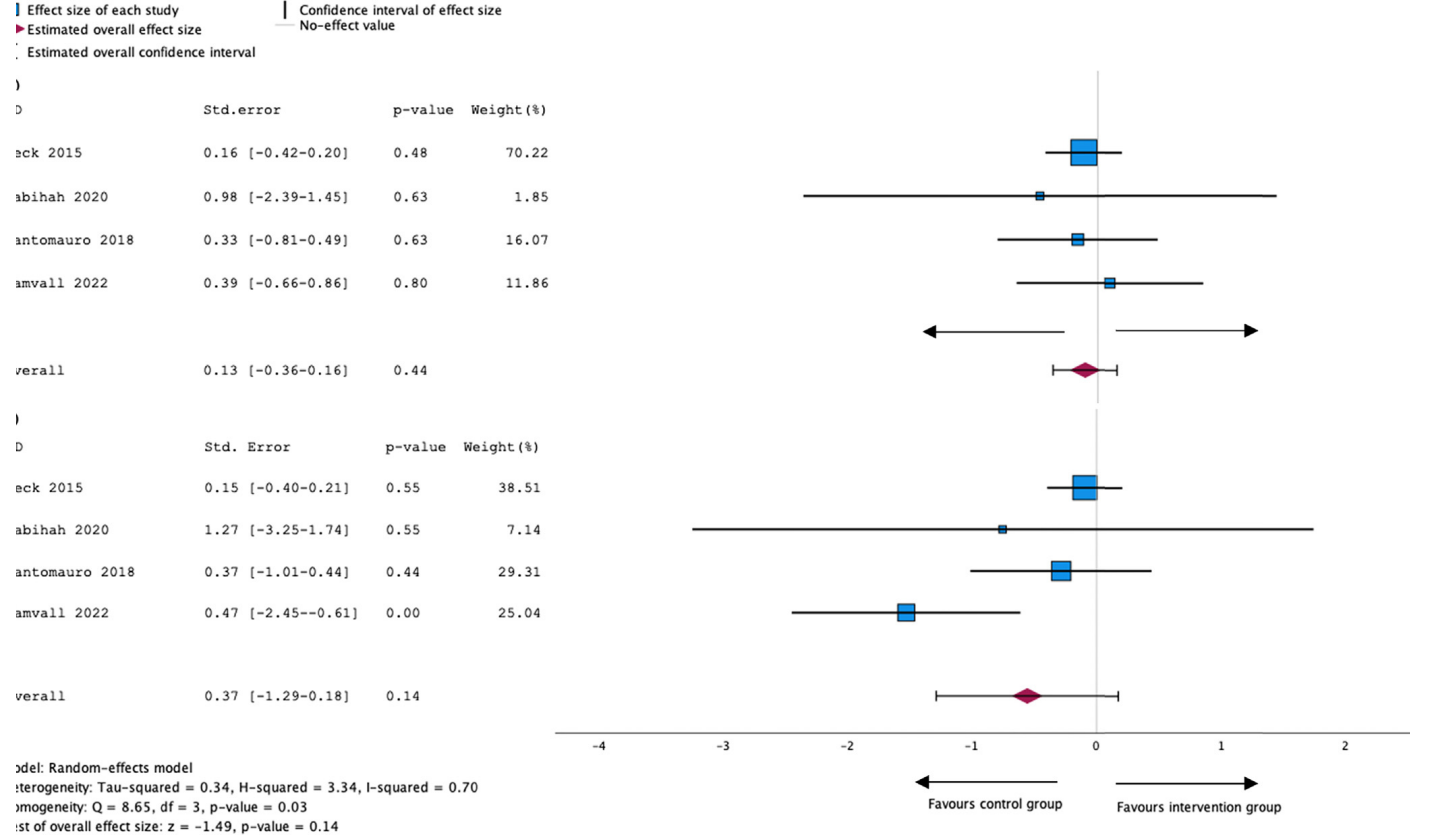
Forest plot depicting size effect calculated with random-effects model for binary data (% students passing the skills test) for the outcomes compression depth (A) and compression rate (B) of the intervention group ‘peer-tutors’. Confidence interval for homogeneity testing with Cochrane’s Q-Test and for the metaanalysis was set at α = 0.05. Included references: Beck 2015^25^, Sabihah 2020^28^, Santomauro 2018^29^, Damvall 2022.^27^

All four studies reported the assessment of chest compression rate and depth at the end-of-course as dichotomous data (% children failing or passing the test).^28^, ^29^ One study^27^ reported the percentage of children fulfilling ERC guideline recommendations, which was rated as ‘pass’. Homogeneity testing for compression rate showed significant heterogeneity (Q = 8.65, df = 3, p-value = 0.03). Data for compression depth showed sufficient homogeneity to calculate the effect size (Q = 0.45, df = 3, p-value = 0.93) which revealed an overall effect of z = −0.77 (p-value = 0.44). Forest plots are depicted in Fig. 2. Due to the small number of studies included in the metanalysis we did not perform a formal assessment of publication bias.^40^

Knowledge and willingness to perform CPR was equivalent for those taught by peer-tutors and by HCPs for short- ^28^ to mid-term.^26^, ^28^

### Schoolteacher-led training compared to training by HCPs (*n* = 7) (Table 4B)

In the five studies comparing schoolteacher led to expert/HCP led training, results were equivalent for all time points and for nearly all skills. 32, 33, 34 Only ‘ventilation volume’ was lower for training by schoolteachers after 2 years in two studies. 30, 35 However, ‘call EMS’, ‘compression depth’, ‘compression rate’, and ‘ventilation rate’ was found better after schoolteacher training. 30, 32, 33, 35 As ‘compression depth’ and ‘compression rate’ were reported with heterogenous outcomes (pass/fail, % correct, performance scores, and definite measurements), a meta-analysis did not appear reasonable. We did not consider further methods to adjust for the inconsistencies between studies.

Schoolteacher training yielded equivalent results to HCPs in three of the five studies for knowledge, willingness to perform CPR and confidence,^30^, ^32^, ^34^ but the other two studies showed HCPs achieved superior results.^35^, ^36^

### Medical students compared to HCP-led training (*n* = 3) (Table 4C)

Results of the overall skills assessment were found to be better after training by medical students than by HCPs, for short- ^31^ and midterm. 31, 37 Regarding specific skills, the studies reported equivalence for early all skills, except for ‘use of defibrillators,’ where the medical students achieved better results than HCPs. Transfer of knowledge for mid- 37, 38 and long-term^38^ was more effective when schoolchildren were instructed by medical students. Self-confidence was reported as equivalent.

## Discussion

Overall, this systematic review showed that for training resuscitation skills, the alternative teachers – peers,^25^, ^26^, ^27^, ^28^, ^29^ schoolteachers^30^, ^31^, ^32^, ^33^, ^34^, ^35^, ^36^ and medical students^31^, ^37^, ^38^, ^41^ – were equivalent to HCPs. Only in one study (with two publications) was the retention of one skill (‘ventilation volume’) lower in the intervention group.^30^, ^35^ Regarding the acquisition of CPR knowledge however, schoolteachers and medical students reached superior results.^35^, ^36^, ^31^, ^32^, ^33^

In the following section, we will discuss the three non-HCP groups in more detail.

### Peer-tutors

The studies included reported that peer teachers achieved equivalent results to HCP instructors for skills, 25, 27, 28, 29 knowledge transfer^26^, ^28^ and confidence boost. ^26^ Peer training has already been shown effective for teenagers in health education, especially when talking about taboo topics. ^42^ A recent study showed that after CPR training, male teenagers were less willing to attempt resuscitation on women. ^43^ Such gender effects when performing CPR in real life have been reported as one factor lowering survival rates of females. 43, 44, 45 A survey of male adults reported that 34% feared sexual harassment charges. ^46^ Issues that can hinder resuscitative efforts might be better addressed with teens by peers than by HCPs. This aspect of training should be further assessed with an appropriate study design.

As a further point, it has been shown that teenagers as peer-tutors who taught health education topics (e.g., healthy nutrition) applied this knowledge in their daily life. ^42^ Likewise, peer-training by medical students has been shown to increase the tutors’ own resuscitation skills, confidence and willingness to initiate CPR. Whether this also applies to schoolchildren is also still unknown.

Studies^25^, ^26^, ^27^, ^28^, ^29^ included here reported heterogeneous preparatory educational strategies for peer-tutors. Peers had received education for between 3 h and 2 days, including either only knowledge and skills of CPR 26, 27, 28, 29 or additional didactic skills such as the Peyton’s four-step approach^25^ or instructions on providing feedback. ^25^ This heterogeneity between studies might explain the divergent results of the peer-taught CPR training. As one example, a study using same-aged peer-tutors reported worse skill acquisition (compared to HCPs) for some skills even after extensive training of the peer-tutors. ^28^ In contrast, another study^25^ intentionally selected peers who were two to three years older than their ‘trainees’ and achieved equivalent results compared to HCPs. However, whether older peers really achieve better results, remains to be determined by specifically designed studies.

### Schoolteacher as trainers

In the studies of this review, training by schoolteachers 30, 31, 32, 33, 34, 35, 36 was overall equivalent 30, 31, 32, 33, 34, 35, 36 to HCP training. For some skills, teachers reached superior results (‘calling EMS’^33^, ‘compression rate^30^, ^35^ and depth^35^′, ‘overall judgement’^32, 33^), while ventilation skills after one year were inferior when taught by teachers^30^, ^35^. Regarding knowledge transfer, teachers were more effective for a variety of outcomes, 30, 32, 34, 35, 36 even for time points up to one-year post-intervention.^30^, ^34^, ^35^, ^36^

The included studies reported initial training of the schoolteachers to become CPR instructors. However, no information was available on whether these teachers were provided with refresher courses, similar to those for ERC-certified BLS instructors. ^47^ Both initial training and refresher courses are important supportive strategies as lack of confidence in their own CPR knowledge and skills has been reported as a relevant factor for lower willingness to teach CPR in schools. ^15^ It has been suggested to add CPR instructor training to the teacher training at universities. ^12^ The impact of subsequent refresher courses has not yet been studied.

### Medical students

Our review indicates that medical students are a good alternative to HCP instructors. 31, 37, 38 Surprisingly, medical students showed superior results in transferring knowledge^37^, ^38^ and in the skills ‘using an AED’^31^ and ‘overall skill assessment’. ^38^ Previous studies have suggested that such training could be incorporated into undergraduate medical curricula considering that acting as a CPR instructor in schools improved medical students’ own CPR skills. 39, 48, 49 Involving medical students in school CPR training programmes was has also been proposed as part of a concept of creating ‘training cascades’, 50, 51 where medical students instruct schoolteachers who then select and train schoolchildren as peer-instructors to teach their classmates. Such a system could demonstrate that essential CPR skills can be acquired by anyone. In addition, university education could be directly linked to a service for the society. However, research is still needed to better understand the effective mechanisms of such cascades.

Overall, this systematic review shows that ‘Kids-Save-Lives’ trainings for schoolchildren does not appear dependent on HCPs as instructors. Schoolteachers have already been proposed by ILCOR and the European Resuscitation Council as equivalent alternatives. 12, 52 This systematic review confirms this recommendation and even suggests that schoolteachers could be the preferred option if given proper training opportunities. For easier implementation, we encourage the inclusion of CPR instructor training into teacher training at university. Such trainings could be held by medical students. Including peer-tutors could help to reach more schoolchildren.

Some important limitations need to be addressed. (1) The overall certainty of evidence was rated from ‘low’ to ‘very low’, even though only controlled studies were included. Reasons included risk of bias, imprecision, and inconsistency. (2) The heterogeneity of the studies included was significant, as the training strategies differed significantly, especially for knowledge transfer (lecture,^25^, ^26^, ^29^, ^30^, ^31^, ^34^, ^35^, ^36^, ^37^, ^38^ e-learning,^27^ video, 28, 29, 32, 34) as well as for study designs (randomized controlled trials, 25, 28, 31, 34 cluster-randomized controlled trials,^32^, ^37^, ^38^ non-randomized experimental studies^26^, ^27^, ^29^, ^30^, ^33^, ^35^, ^36^). Additional subgrouping of the studies according to short-term,^25^, ^27^, ^28^, ^32^, ^33^, ^34^ mid-term^26^, ^27^, ^30^, ^31^, ^32^, ^34^, ^35^, ^36^, ^37^, ^38^ and long-term-30, 35 retention further lowered the sample sizes. A further source of heterogeneity was the large variation in assessment tools, including individually created tools that had not been adequately validated. Even for rather objective skill assessments, some authors reported data retrieved from manikins^27^, ^35^, ^30^, ^31^ while others used individually created checklists.^25^, ^28^, ^29^, ^31^, ^32^, ^33^, ^34^ Analysis of more abstract outcomes, such as willingness to perform CPR^26^, ^34^ and confidence,^30^, ^35^, ^38^ was also performed based on individually-designed questionnaires, which again were often not validated. In addition, we did not find information on whether questionnaires had been adapted to the schoolchildren’s ages.

As a further limitation, publication bias is likely given that the investigators were interested in the topic.^53^ However, we did not formally assess publication bias as only a few studies could be included in the *meta*-analyses. Finally, the included studies were performed in Europe and two in Asia. Whether these findings can be transferred to other countries with different cultural and/or socioeconomic contexts cannot be answered with certainty.

### Knowledge gaps and future research

Several open questions remain regarding teachers in ‘Kids-Save-Lives’ trainings. For peer-tutors, a small age gap between tutor and trainee may benefit schoolchildren, but this hypothesis requires further investigation. For both peer-tutors and schoolteachers, clearer information is needed about refresher courses, including the extent, content, and timing of such courses. Studies are also needed to evaluate combinations of peer-training and schoolteacher-led training, as well as ‘training cascades’ with medical students.

To allow for appropriate comparisons and meta-analyses, validated age-adapted training programmes, questionnaires, and skills-assessments need to be standardized. Therefore, uniform reporting guidelines for educational studies in resuscitation are urgently needed.

## Conclusions

This systematic review analysed studies comparing different alternative training personnel (peer-tutors, schoolteachers, or medical students) with HCPs as instructors. Overall, the studies showed equivalent learning outcomes for CPR skills and knowledge up to one year, and some aspects of knowledge acquisition were even superior after training by non-HCPs. For peer training, a small age gap between peers and the trainees trained might be advisable. Based on these results, future studies should address whether involving non-HCP instructors makes these programs more cost-effective and easier to integrate into school curricula, as well as examining which type of refresher courses are most effective for various non-HCP instructor groups. This would potentially increase the overall effectiveness of Kids-Save-Lives programmes.

## Ethical approval

Not applicable.

## Funding

None (academic study).

## CRediT authorship contribution statement

**A. Mollo:** Writing – original draft, Software, Project administration, Methodology, Investigation, Formal analysis, Data curation. **S. Beck:** Writing – review & editing, Investigation, Formal analysis, Data curation. **A. Degel:** Writing – review & editing, Investigation, Formal analysis. **R. Greif:** Writing – review & editing, Methodology, Investigation, Formal analysis. **J. Breckwoldt:** Writing – review & editing, Writing – original draft, Validation, Supervision, Resources, Methodology, Investigation, Formal analysis, Data curation, Conceptualization.

## Declaration of competing interest

The authors declare the following financial interests/personal relationships which may be considered as potential competing interests: [The authors declare the following financial interests/personal relationships which may be considered as potential competing interests: **AM**: None to declare. **SB**: declared an intellectual conflict of interest and was excluded from data extraction and Risk of Bias assessment of the studies she co-authored.^25^, ^39^ **AD**: None to declare. **RG**: European Resuscitation Council Director of Guidelines and ILCOR, ILCOR Task Force Chair Education, Implementation and Teams. RG is an Editorial Board member of ‘Resuscitation Plus’. **JB**: Member of the ILCOR EIT Task Force (Education, Implementation and Teams); member of the ERC Science and Education Committee /SEC IES; member of the writing group for the ERC Guidelines 2025 (Chapter Education).].

## References

[1] Olasveengen T.M., Semeraro F., Ristagno G. (2021). European Resuscitation Council Guidelines 2021: Basic Life Support. Resuscitation.

[2] Lindner T.W., Søreide E., Nilsen O.B., Torunn M.W., Lossius H.M. (2011). Good outcome in every fourth resuscitation attempt is achievable–an Utstein template report from the Stavanger region. Resuscitation.

[3] Kragholm K., Wissenberg M., Mortensen R.N. (2017). Bystander efforts and 1-year outcomes in out-of-hospital cardiac arrest. N Engl J Med.

[4] Gräsner J.T., Wnent J., Herlitz J. (2020). Survival after out-of-hospital cardiac arrest in Europe - Results of the EuReCa TWO study. Resuscitation.

[5] Hasselqvist-Ax I., Riva G., Herlitz J. (2015). Early cardiopulmonary resuscitation in out-of-hospital cardiac arrest. N Engl J Med.

[6] Yan S., Gan Y., Jiang N. (2020). The global survival rate among adult out-of-hospital cardiac arrest patients who received cardiopulmonary resuscitation: a systematic review and meta-analysis. Crit Care.

[7] Wagner P., Schloesser S., Braun J., Arntz H.R., Breckwoldt J. (2020). In out-of-hospital cardiac arrest, is the positioning of victims by bystanders adequate for CPR? A cohort study. BMJ Open.

[8] Breckwoldt J., Schloesser S., Arntz H.R. (2009). Perceptions of collapse and assessment of cardiac arrest by bystanders of out-of-hospital cardiac arrest (OOHCA). Resuscitation.

[9] Strömsöe A., Andersson B., Ekström L. (2010). Education in cardiopulmonary resuscitation in Sweden and its clinical consequences. Resuscitation.

[10] Bohn A., Lukas R.P., Breckwoldt J., Bottiger B.W., Van Aken H. (2015). 'Kids save lives': why schoolchildren should train in cardiopulmonary resuscitation. Curr Opin Crit Care.

[11] Böttiger B.W., Van Aken H. (2015). Kids save lives–Training school children in cardiopulmonary resuscitation worldwide is now endorsed by the World Health Organization (WHO). Resuscitation.

[12] Schroeder D.C., Semeraro F., Greif R. (2023). KIDS SAVE LIVES: basic life support education for schoolchildren: a narrative review and scientific statement from the International Liaison Committee on Resuscitation. Circulation.

[13] Schroeder D.C., Semeraro F., Greif R. (2023). KIDS SAVE LIVES: basic life support education for schoolchildren: a narrative review and scientific statement from the International Liaison Committee on Resuscitation. Resuscitation.

[14] Semeraro F., Greif R., Böttiger B.W. (2021). European Resuscitation Council Guidelines 2021: Systems saving lives. Resuscitation.

[15] Wingen S., Jeck J., Schroeder D.C., Wingen-Heimann S.M., Drost R., Böttiger B.W. (2022). Facilitators and barriers for the implementation of resuscitation training programmes for schoolchildren: A systematic review. Eur J Anaesthesiol.

[16] Greif R., Lockey A., Breckwoldt J. (2021). European Resuscitation Council Guidelines 2021: education for resuscitation. Resuscitation.

[17] Greif R., Bhanji F., Bigham B.L. (2020). Education, Implementation, and Teams: 2020 international consensus on cardiopulmonary resuscitation and emergency cardiovascular care science with treatment recommendations. Circulation.

[18] Greif R., Bhanji F., Bigham B.L. (2020). Education, Implementation, and Teams: 2020 international consensus on cardiopulmonary resuscitation and emergency cardiovascular care science with treatment recommendations. Resuscitation.

[19] Page M.J., McKenzie J.E., Bossuyt P.M. (2021). The PRISMA 2020 statement: an updated guideline for reporting systematic reviews. BMJ.

[20] Sterne J.A., Hernán M.A., Reeves B.C. (2016). ROBINS-I: a tool for assessing risk of bias in non-randomised studies of interventions. BMJ.

[21] Sterne J.A.C., Savović J., Page M.J. (2019). RoB 2: a revised tool for assessing risk of bias in randomised trials. BMJ.

[22] Higgins JPT TJ, Chandler J, Cumpston M, Li T, Page MJ, Welch VA. Cochrane Handbook for Systematic Reviews of Interventions http://www.training.cochrane.org/handbook2023 [updated August 2023.

[23] Campbell M., McKenzie J.E., Sowden A. (2020). Synthesis without meta-analysis (SWiM) in systematic reviews: reporting guideline. BMJ.

[24] Haidich A.B. (2010). Meta-analysis in medical research. Hippokratia.

[25] Beck S., Issleib M., Daubmann A., Zollner C. (2015). Peer education for BLS-training in schools? Results of a randomized-controlled, noninferiority trial. Resuscitation.

[26] Choi H.S., Lee D.H., Kim C.W., Kim S.E., Oh J.H. (2015). Peer-assisted learning to train high-school students to perform basic life-support. World J Emerg Med.

[27] Damvall D.A., Birkenes T.S., Nilsen K., Haaland S.H., Myklebust H., Nordseth T. (2022). Can high school students teach their peers high quality cardiopulmonary resuscitation (CPR)?. Resusc plus.

[28] Sabihah A., Shamsuriani M.J., Fadzlon M.Y. (2020). Peer trainers compared with basic life support trainers in delivering effective cardiopulmonary resuscitation training to secondary school students. Medicine & Health (Universiti Kebangsaan Malaysia)..

[29] Santomauro M.V.I., Riganti C., Palma G. (2018). Future perspective in BLSD training: the importance of peer-to peer education in high school students. J Transl Sci.

[30] Bohn A., Van Aken H.K., Mollhoff T. (2012). Teaching resuscitation in schools: annual tuition by trained teachers is effective starting at age 10. A four-year prospective cohort study. Resuscitation..

[31] Cuijpers P.J., Bookelman G., Kicken W., de Vries W., Gorgels A.P. (2016). Medical students and physical education students as CPR instructors: an appropriate solution to the CPR-instructor shortage in secondary schools?. Neth Hear J.

[32] Lanzas D., Nunes P., Perelman J. (2022). Training program in resuscitation maneuvers delivered by teachers in a school setting: an economic argument. Rev Port Cardiol.

[33] Pérez-Bailón A.M., Parrilla-Ruiz F.M., Gómez-Moreno G., Herrera-Mingorance J.D., Cárdenas-Cruz A. (2023). Comparative study on the role of the secondary school teacher as the key for life support training: Cervantes Model. Educacion Medica.

[34] Yeung C.Y., So K.Y., Cheung H.H.T., Hou P.Y., Ko H.F., Lee A. (2023). Comparison of instructor-led compression-only cardiopulmonary resuscitation and automated external defibrillator training for secondary school students: a multicenter noninferiority randomized trial. Resuscitation plus..

[35] Lukas R.P., Van Aken H., Molhoff T. (2016). Kids save lives: a six-year longitudinal study of schoolchildren learning cardiopulmonary resuscitation: who should do the teaching and will the effects last?. Resuscitation.

[36] Jimenez-Fabrega X., Escalada-Roig X., Miro O. (2009). Comparison between exclusively school teacher-based and mixed school teacher and healthcare provider-based programme on basic cardiopulmonary resuscitation for secondary schools. Emerg Med J.

[37] Dîrzu D.S., Hagău N., Boț T., Fărcaș L., Copotoiu S.M. (2018). Training in cardiopulmonary resuscitation provided by medical students, residents and specialists: a non-inferiority trial. Hong Kong J Emerg Med.

[38] Haseneder R., Skrzypczak M., Haller B. (2019). Impact of instructor professional background and interim retesting on knowledge and self-confidence of schoolchildren after basic life support training: a cluster randomised longitudinal study. Emerg Med J.

[39] Beck S., Meier-Klages V., Michaelis M. (2016). Teaching school children basic life support improves teaching and basic life support skills of medical students: a randomised, controlled trial. Resuscitation.

[40] Mavridis D., Salanti G. (2014). How to assess publication bias: funnel plot, trim-and-fill method and selection models. Evid Based Ment Health.

[41] Cuijpers P., Schakelaar E., Van Merrienboer J., Gorgels A. (2011). Three instructional methods for CPR training in secondary schools: practical training remains necessary. Resuscitation.

[42] Tolli M.V. (2012). Effectiveness of peer education interventions for HIV prevention, adolescent pregnancy prevention and sexual health promotion for young people: a systematic review of European studies. Health Educ Res.

[43] Wingen S., Ecker H., Schroeder D.C., Bartholme B., Bottiger B.W., Wetsch W.A. (2022). Addressing the helper's and victim's gender is crucial in schoolchildren resuscitation training-A prospective, educative interventional trial. J Clin Med.

[44] Blom M.T., Oving I., Berdowski J., van Valkengoed I.G.M., Bardai A., Tan H.L. (2019). Women have lower chances than men to be resuscitated and survive out-of-hospital cardiac arrest. Eur Heart J.

[45] Ok Ahn K., McNally B., Al-Araji R., Cisneros C., Chan P.S. (2023). Sex differences in the association between bystander CPR and survival for Out-of-Hospital cardiac arrest. Resuscitation.

[46] Lubrano R., Romero S., Scoppi P. (2005). How to become an under 11 rescuer: a practical method to teach first aid to primary schoolchildren. Resuscitation.

[47] ERC. Available from: https://www.erc.edu/courses/basic-life-support.

[48] Breckwoldt J., Beetz D., Schnitzer L., Waskow C., Arntz H.R., Weimann J. (2007). Medical students teaching basic life support to school children as a required element of medical education: a randomised controlled study comparing three different approaches to fifth year medical training in emergency medicine. Resuscitation.

[49] Baldi E., Savastano S., Contri E. (2020). Mandatory cardiopulmonary resuscitation competencies for undergraduate healthcare students in Europe: a European Resuscitation Council guidance note. Eur J Anaesthesiol.

[50] Connolly M., Toner P., Connolly D., McCluskey D.R. (2007). The 'ABC for life' programme - teaching basic life support in schools. Resuscitation.

[51] Mc Grath P., Laverty L., Toner P., Connolly M. (2012). ABC for Life: a Programme of BLS training to primary school children in Northern Ireland Since 2004. Irish J Med Sci.

[52] Bottiger B.W., Lockey A., Georgiou M. (2020). KIDS SAVE LIVES: ERC Position statement on schoolteachers' education and qualification in resuscitation. Resuscitation.

[53] Murad M.H., Chu H., Lin L., Wang Z. (2018). The effect of publication bias magnitude and direction on the certainty in evidence. BMJ Evid Based Med.

