# Supplementary material for: Kids save lives: Who should train schoolchildren in resuscitation? A systematic review
Source: Resusc Plus. 2024 Aug 29;20:100755. doi: 10.1016/j.resplu.2024.100755 (PMC11401354; doi:10.1016/j.resplu.2024.100755)
Supplement: Supplementary Data 1 [file mmc1.docx]

**Supplementary material**

| **Peer-led training** | **Study** | **Training duration and strategy** | **Instructors' training** |
| --- | --- | --- | --- |
|  | Beck^(^[^25^](#_ENREF_25)^)^ | 3h: 30 min theory (lecture), 75 min skills training (manikin training, demonstration) | 2x 3h by two anesthesiologists knowledge of OHCA, BLS training, four-step-approach, how to give feedback |
|  | Choi^(^[^26^](#_ENREF_26)^)^ | 1h (demonstration, manikin training) | 3h by BLS specialist |
|  | Damvall^(^[^27^](#_ENREF_27)^)^ | Theory (e-learning), 90 min during class (skills training) | 2d by NRC-certified instructor knowledge and skills training |
|  | Sabihah^(^[^28^](#_ENREF_28)^)^ | 8min theory (video), skills training | 1d by AHA-certified instructor |
|  | Santomauro^(^[^29^](#_ENREF_29)^)^ | 6h: 2h theory (lecture, videos), skills training | 2d CPR instructor course |
| **schoolteacher-led training** | Bohn^(^[^30^](#_ENREF_30)^)^ | 1h theory (lecture), 2h skills training | 1h course by emergency physicians |
|  | Cuijpers^(^[^31^](#_ENREF_31)^)^ | 90 min (demonstration, skills training) | course by DRC-certified instructors |
|  | Jimenez-Fabrega^(^[^36^](#_ENREF_36)^)^ | 7x1h (lecture, skills training) | AHA instructor course |
|  | Lanzas^(^[^32^](#_ENREF_32)^)^ | 16 min theory (video), 5 min skills training | AHA BLS course + specific training program for instructions in schools |
|  | Lukas^(^[^35^](#_ENREF_35)^)^ | 1h theory (lecture), 2h skills training | 1h course by emergency physicians |
|  | Perez-Bailon^(^[^33^](#_ENREF_33)^)^ | *NI* | *NI* |
|  | Yeung^(^[^34^](#_ENREF_34)^)^ | 2h (lecture, video, skill training) | teachers had AHA BLS provider certificate; received materials from "CPR in school training kit"; briefing before training |
| **med. Students led training** | Cuijpers^(^[^31^](#_ENREF_31)^)^ | 90 min (demonstration, skills training) | course by DRC-certified instructors |
|  | Dirzu^(^[^37^](#_ENREF_37)^)^ | 1h theory, and 1 week later skills training | training by anesthesiologist |
|  | Haseneder^(^[^38^](#_ENREF_38)^)^ | 90 min (lecture, demonstration, skills training) | no specifical training before course as |

Supplemental Table 1: Overview of training duration and strategy for the schoolchildren and the instructors’ training.

| **GRADE judgement** | | | | | | | | | | | |
| --- | --- | --- | --- | --- | --- | --- | --- | --- | --- | --- | --- |
| **Subgroup** | **Outcomes** | **Risk of bias** | **Imprecision** | **Inconsistency** | **Indirectness** | **Publication bias** | **Effect magnitude** | **Plausible confounding** | **dose-response gradient** | **Overall** |  |
| **Peer-tutor (n= 5)** | Skills (n=4)^(^[^25^](#_ENREF_25)^,^ [^27-29^](#_ENREF_27)^)^ | Low-Moderate | -1 | -1 | 0 | -1 | - | - | - | Very low |  |
|  | Knowledge (n =2)^(^[^26^](#_ENREF_26)^,^ [^28^](#_ENREF_28)^)^ | Low-Serious | -1 | -1 | 0 | 0 | - | - | - | Very low |  |
|  | Willingness (n=1)^(^[^26^](#_ENREF_26)^)^ | Serious | -1 | -1 | 0 | 0 | - | - | - | Very low |  |
| **Schoolteacher (n=7)** | Skills (n=5)^(^[^30-33^](#_ENREF_30)^,^ [^35^](#_ENREF_35)^)^ | Low-Serious | -1 | -1 | -1 | -1 | - | - | - | Very low |  |
|  | Knowledge (n =5)^(^[^30^](#_ENREF_30)^,^ [^32^](#_ENREF_32)^,^ [^34-36^](#_ENREF_34)^)^ | Low-Serious | -1 | -1 | -1 | -1 | - | - | - | Very low |  |
|  | Confidence (n=2)^(^[^30^](#_ENREF_30)^,^ [^35^](#_ENREF_35)^)^ | Serious | -1 | -1 | -1 | 0 | - | - | - | Very low |  |
|  | Willingness (n=1)^(^[^34^](#_ENREF_34)^)^ | Low | -1 | -1 | -1 | 0 | - | - | - | Very low |  |
| **Medical students (n=3)** | Skills (n=2)([31](#_ENREF_31), [37](#_ENREF_37)) | Moderate | -1 | -1 | 0 | 0 | - | - | - | Low |  |
|  | Knowledge (n=2)^(^[^37^](#_ENREF_37)^,^ [^38^](#_ENREF_38)^)^ | Moderate | -1 | -1 | 0 | 0 | - | - | - | Low |  |
|  | Confidence (n=1)^(^[^38^](#_ENREF_38)^)^ | Moderate | -1 | -1 | 0 | 0 | - | - | - | Low |  |

Supplemental Table 2: Assessment of Certainty of Evidence with the GRADE methodology
